# Supplementary material for: Programming Mechanism of Adipose Tissue Expansion in the Rat Offspring of Obese Mothers Occurs in a Sex-Specific Manner
Source: Nutrients. 2023 May 9;15(10):2245. doi: 10.3390/nu15102245 (PMC10223280; doi:10.3390/nu15102245)
Supplement: Supplementary file 1 [file nutrients-15-02245-s001.zip › nutrients-2306454-supplementary.pdf]

Supplementary Material

Table S1. Target genes included in the Adipogenesis qPCR array (Qiagen, RT[2] Adipogenesis Profiler PARN-049Z #330231).

Gene Symbol	RefSeq	Description
Acacb	NM_053922	Acetyl-Coenzyme A carboxylase ß
Adipoq	NM_144744	Adiponectin, C1Q and collagen domain containing
Adrb2	NM_012492	Adrenoceptor ß-2
Agt	NM_134432	Angiotensinogen
Angpt2	NM_134454	Angiopoietin 2
Axin1	NM_024405	Axin 1
Bmp2	NM_017178	Bone morphogenetic protein 2
Bmp4	NM_012827	Bone morphogenetic protein 4
Bmp7	NM_001191856	Bone morphogenetic protein 7
Ccnd1	NM_171992	Cyclin D1
Cdk4	NM_053593	Cyclin-dependent kinase 4
Cdkn1a	NM_080782	Cyclin-dependent kinase inhibitor 1A
Cdkn1b	NM_031762	Cyclin-dependent kinase inhibitor 1B
Cebpa	NM_012524	CCAAT/enhancer binding protein (C/EBP), α
Cebpb	NM_024125	CCAAT/enhancer binding protein (C/EBP), ß
Cebpd	NM_013154	CCAAT/enhancer binding protein (C/EBP), δ
Cfd	NM_001077642	Complement factor D (adipsin)
Creb1	NM_031017	cAMP responsive element binding protein 1
Ddit3	NM_024134	DNA-damage inducible transcript 3
Dio2	NM_031720	Deiodinase, iodothyronine, type II
Dkk1	NM_001106350	Dickkopf homolog 1
Dlk1	NM_053744	Delta-like 1 homolog
E2f1	NM_001100778	E2F transcription factor 1
Egr2	NM_053633	Early growth response 2
Fabp4	NM_053365	Fatty acid binding protein 4
Fasn	NM_017332	Fatty acid synthase
Fgf1	NM_012846	Fibroblast growth factor 1
Fgf10	NM_012951	Fibroblast growth factor 10
Fgf2	NM_019305	Fibroblast growth factor 2
Fos	NM_022197	FBJ osteosarcoma oncogene
Foxc2	NM_001101680	Forkhead box C2

Table S1. Target genes included in the Adipogenesis qPCR array (Qiagen, RT^2 Adipogenesis Profiler PARN-049Z #330231). Continued.

Gene Symbol	RefSeq	Description
Gata2	NM_033442	GATA binding protein 2
Gata3	NM_133293	GATA binding protein 3
Hes1	NM_024360	Hes family bHLH transcription factor 1
Insr	NM_017071	Insulin receptor
Irs1	NM_012969	Insulin receptor substrate 1
Irs2	NM_001168633	Insulin receptor substrate 2
Jun	NM_021835	Jun oncogene
Klf15	NM_053536	Kruppel-like factor 15
Klf2	NM_001007684	Kruppel-like factor 2
Klf3	NM_001105742	Kruppel-like factor 3
Klf4	NM_053713	Kruppel-like factor 4
Lep	NM_013076	Leptin
Lipe	NM_012859	Lipase, hormone sensitive
Lmna	NM_001002016	Lamin A
Lpl	NM_012598	Lipoprotein lipase
Lrp5	NM_001106321	Low density lipoprotein receptor-related protein 5
Mapk14	NM_031020	Mitogen activated protein kinase 14
Ncoa2	NM_031822	Nuclear receptor coactivator 2
Ncor1	NM_001271103	Nuclear receptor co-repressor 1
Ncor2	NM_001108334	Nuclear receptor co-repressor 2
Nr0b2	NM_057133	Nuclear receptor subfamily 0, group B, member 2
Nr1h3	NM_031627	Nuclear receptor subfamily 1, group H, member 3
Nrf1	NM_001100708	Nuclear respiratory factor 1
Ppara	NM_013196	Peroxisome proliferator activated receptor α
Ppard	NM_013141	Peroxisome proliferator activated receptor δ
Pparg	NM_013124	Peroxisome proliferator activated receptor γ
Ppargc1a	NM_031347	Peroxisome proliferator activated receptor γ, coactivator 1 α
Ppargc1b	NM_176075	Peroxisome proliferatoractivated receptor γ, coactivator 1 ß
Rb1	NM_017045	Retinoblastoma 1
Retn	NM_144741	Resistin
Runx1t1	NM_001108657	RUNX1 partner transcriptional co-repressor 1
Rxra	NM_012805	Retinoid X receptor α
Sfrp1	NM_001276712	Secreted frizzled-related protein 1
Sfrp5	NM_001107591	Secreted frizzled-related protein 5

Table S1. Target genes included in the Adipogenesis qPCR array (Qiagen, RT[2] Adipogenesis Profiler PARN-049Z #330231). Continued.

Gene Symbol	RefSeq	Description
Shh	NM_017221	Sonic hedgehog
Sirt1	NM_001107627	Sirtuin 1
Sirt2	NM_001008368	Sirtuin 2
Sirt3	NM_001106313	Sirtuin 3
Slc2a4	NM_012751	Solute carrier family 2 member 4
Src	NM_031977	SRC proto-oncogene, non-receptor tyrosine kinase
Srebf1	NM_001276707	Sterol regulatory element binding transcription factor 1
Stat5a	NM_017064	Signal transducer and activator of transcription 5A
Taz	NM_001025748	Tafazzin
Tcf7l2	NM_001191052	Transcription factor 7-like 2
Tsc22d3	NM_031345	TSC22 domain family, member 3
Twist1	NM_053530	Twist homolog 1
Ucp1	NM_012682	Uncoupling protein 1
Vdr	NM_017058	Vitamin D receptor
Wnt1	NM_001105714	Wnt family member 1
Wnt10b	NM_001108111	Wnt family member 10B
Wnt3a	NM_001107005	Wnt family member 3A
Wnt5a	NM_022631	Wnt family member 5A
Wnt5b	NM_001100489	Wnt family member 5B

Table S2. Gene expression related to adipogenesis in F1C female vs. F1C male, F1MO vs. F1C male, and F1MO vs. F1C female. Fold regulation and their statistical significance are shown by P-value.

Gene	Female C vs Male C		Male MO vs Male C		Female MO vs Female C	
	Fold Regulation	P-value (t-test)	Fold Regulation	P-value (t-test)	Fold Regulation	P-value (t-test)
Acacb	-7.13	0.001937	-11.68	0.005559	-1.34	0.81152
Adipoq	-1.66	0.004138	-1.42	0.057329	-2.77	0.425877
Adrb2	-18.28	0.003532	-10.35	0.016083	-1.48	0.831162
Agt	-94.78	0.000185	-23.63	0.000553	-1.43	0.472237
Angpt2	-4.77	0.000497	-10.02	0.001452	-1.27	0.650867
Axin1	-6.31	0.003201	-6.81	0.007737	-1.76	0.521741
Bmp2	-34.23	0.00003	-13.76	0.008233	-1.28	0.543646
Bmp4	-84.12	0.011465	-10.35	0.132314	3.39	0.360335
Bmp7	-5.93	0.014906	-2.66	0.18733	-1.99	0.685539
Ccnd1	-19.88	0.001497	-15.8	0.007551	-2.29	0.879968
Cdk4	-2.35	0.010059	-4.28	0.003787	-1.77	0.345884
Cdkn1a	1.32	0.378179	1.78	0.112561	-1.82	0.234703
Cdkn1b	-9.22	0.000326	-17.78	0.008779	-1.28	0.955673
Cebpa	-10.58	0.000041	-7.51	0.001577	-2.69	0.86901
Cebpb	-7.68	0.162938	-6.07	0.069391	1.02	0.599995
Cebpd	-6.97	0.012672	-12.98	0.016645	-1.46	0.821398
Cfd	-1.88	0.038247	-1.95	0.013456	-2.19	0.470085
Creb1	-2.76	0.022961	-4.23	0.010704	-1.13	0.680954
Ddit3	-7.26	0.00013	-3.59	0.06208	-1.82	0.928194
Dio2	1.11	0.78039	-4	0.08139	-1.98	0.3954
Dkk1	5.07	0.350605	1.88	0.34757	-1.23	0.567709
Dlk1	4.99	0.345871	1.17	0.406364	-1.41	0.444039
E2f1	-31.85	0.004625	-12.92	0.026269	-1.64	0.450255
Egr2	-1.19	0.390536	-7.17	0.192504	-7.63	0.006147
Fabp4	-1.4	0.16247	-1.11	0.595381	-2.63	0.242143
Fasn	-29.13	0.000036	-14.15	0.000413	-3.95	0.480093
Fgf1	-3.61	0.23968	-3.24	0.231989	-1.64	0.303775
Fgf10	-3.96	0.001047	-5.88	0.00729	-2.31	0.780402
Fgf2	-4.06	0.000283	-4.18	0.004049	-2.05	0.407873
Fos	-69.39	0.037006	-3.01	0.938968	2.77	0.191962
Foxc2	-1.58	0.221158	-2.39	0.235602	1.14	0.734463
Gata2	-3.93	0.007985	-10.34	0.011506	-1.74	0.461775

Table S2. Gene expression related to adipogenesis in F1C female vs. F1C male, F1MO vs. F1C male, and F1MO vs. F1C female. Fold regulation and their statistical significance are shown by P-value. Continued.

**Gene**	**Female C vs Male C**		**Male MO vs Male C**		**Female MO vs Female C**	
	Fold Regulation	P-value (t-test)	Fold Regulation	P-value (t-test)	Fold Regulation	P-value (t-test)
Gata3	-2.17	0.648843	-4.4	0.014576	2.03	0.683673
Hes1	1.63	0.322541	-1.04	0.93821	-1.45	0.391768
Insr	-27.91	0.000058	-25.39	0.002376	-1.38	0.75383
Irs1	-1.96	0.098727	-3.74	0.027274	-2.24	0.287748
Irs2	-6.05	0.002799	-7.65	0.005831	-1.18	0.887381
Jun	-16.66	0.000548	-6.33	0.035795	-6.09	0.792149
Klf15	-12.62	0.000772	-9.24	0.00914	-1.91	0.579175
Klf2	-10.28	0.063535	-18.68	0.077231	-1.28	0.674014
Klf3	-4.77	0.000299	-4.34	0.021837	-1.21	0.608617
Klf4	-9.49	0.004401	-2.32	0.148705	-2.06	0.950436
Lep	-241.77	0.007155	-21.28	0.035538	-1.36	0.317598
Lipe	-11.22	0.000012	-12.91	0.00043	-1.72	0.68677
Lmna	-3.94	0.00626	-7.09	0.012844	-1.61	0.417597
Lpl	-12.42	0.000022	-5.6	0.012988	-1.56	0.668699
Lrp5	-23.06	0.000456	-22.34	0.001742	-2.46	0.879182
Mapk14	-5.02	0.00003	-5.49	0.000644	-1.18	0.908347
Ncoa2	-30.26	0.000279	-8.29	0.025738	1.82	0.578184
Ncor1	-5.2	0.000089	-6.04	0.001808	-1.14	0.903219
Ncor2	-3.31	0.103843	-4.79	0.06552	-1.63	0.355795
Nr0b2	1.34	0.369604	-1.53	0.973038	-1.05	0.48714
Nr1h3	-12.41	0.000088	-13.36	0.00084	-1.23	0.717276
Nrf1	-7.84	0.003213	-10.03	0.007579	-1.19	0.931078
Ppara	-13.25	0.000107	-6.08	0.001459	-1.19	0.613562
Ppard	-16.82	0.000373	-12.12	0.003919	1.2	0.770618
Pparg	-3.47	0.000159	-1.72	0.076616	-2.23	0.89316
Ppargc1a	-7.36	0.031431	-3.15	0.01838	2.39	0.534026
Ppargc1b	-5.53	0.000442	-13.31	0.00439	1.11	0.660308
Rb1	-4.28	0.000138	-3.79	0.00634	-1.1	0.854724
Retn	-65.35	0.000001	-7.17	0.000904	-3.25	0.756083
Runx1t1	-23.03	0.033968	-9.62	0.081178	1.6	0.98338
Rxra	-3.33	0.001114	-5.63	0.002687	-1.56	0.459988
Sfrp1	-4.26	0.025049	-3.69	0.126217	1.67	0.427995

Table S2. Gene expression related to adipogenesis in F1C female vs. F1C male, F1MO vs. F1C male and F1MO vs. F1C female. Fold regulation and their statistical significance are shown by P-value. Continued.

**Gene**	**Female C vs Male C**		**Male MO vs Male C**		**Female MO vs Female C**	
	Fold Regulation	P-value (t-test)	Fold Regulation	P-value (t-test)	Fold Regulation	P-value (t-test)
Sfrp5	-9.58	0.008733	-16.78	0.007086	1.7	0.313974
Shh	2.9	0.086051	1.09	0.584828	-1.71	0.18707
Sirt1	-3.44	0.000617	-3.74	0.008601	1.02	0.943073
Sirt2	-4.22	0.000324	-4.12	0.006598	-1.69	0.843386
Sirt3	-131.77	0.000064	-20.6	0.016726	1.73	0.464262
Slc2a4	-44.88	0.000001	-30.93	0.00011	-1	0.403149
Src	-20.16	0.003179	-25.12	0.007971	-1.61	0.637139
Srebf1	-22.01	0.012492	-29.61	0.01769	-3.27	0.808335
Stat5a	-17.95	0.000711	-18.34	0.002974	1.81	0.522744
Taz	-3.14	0.000036	-4.58	0.002536	-1.07	0.866658
Tcf7l2	-3.76	0.000234	-4.61	0.005572	-1.56	0.830986
Tsc22d3	-14.34	0.000051	-11.63	0.003807	1.09	0.394781
Twist1	-10.83	0.003292	-9.28	0.010923	-1.85	0.975892
Ucp1	2.58	0.366505	1.23	0.62553	-1.11	0.819512
Vdr	-15.47	0.000938	-12.01	0.002436	-1.19	0.510683
Wnt1	1.56	0.931468	1.08	0.928067	-1.71	0.18707
Wnt10b	-36.56	0.0072	-22.73	0.024746	-1.71	0.18707
Wnt3a	-1.02	0.722307	-2.06	0.202856	1.71	0.254456
Wnt5a	-14.53	0.000919	-13.8	0.007067	-3.98	0.667965
Wnt5b	-7.99	0.043414	-22.79	0.007034	3.59	0.257902
